# SR140333 counteracts NK-1 mediated cell proliferation in human breast cancer cell line T47D

**DOI:** 10.1186/1756-9966-29-55

**Published:** 2010-05-24

**Authors:** Wei-Qing Huang, Ji-Gang Wang, Lei Chen, Hong-Jun Wei, Hua Chen

**Affiliations:** 1 Department of Pathology, Qingdao Municipal Hospital, 5 Donghai Middle Road, Qingdao, Shandong, 266071, China; 2 Department of Pathology, The Affiliated Hospital of Medical College, Qingdao University, 16 Jiangsu Road, Qingdao, Shandong, 266003, China; 3 Department of Physiology, Qingdao University Medical College, 308 Ningxia Road, Qingdao, Shandong, 266071, China

## Abstract

**Background:**

It has been demonstrated that certain NK-1 antagonists could reduce proliferation of several cancer cell lines, however, it is unknown whether SR140333 exerts proliferation inhibition in breast cancer cell line.

**Methods:**

Immunohistochemical staining was carried out to investigate the immunolocation of NK-1 in breast cancer tissues and T47D cell line, thereafter, various concentrations of [Sar9, Met(O2)11]substance P and SR140333 were applied alone or combined. MTT assay was applied to detect cytoactivation and coulter counter was to detect growth curve. The Hoechst33258 staining was performed to detect apoptosis.

**Results:**

We found that breast cancer and T47D cells bear positive expression of NK-1. SR140333 inhibited cell growth in a dose dependent manner. Furthermore, SR140333 could counteract [Sar9, Met(O2)11]substance P induced proliferation. Hoechst33258 staining revealed the presence of apoptosis after SR140333 treatment.

**Conclusions:**

Our study demonstrated SR140333 exert proliferation inhibition in breast cancer cell line T47D and indicates NK-1 play a central role in the substance P related cell proliferation in breast cancer.

## Background

Breast cancer is the most common cancer among women worldwide. It already is an urgent public health problem in high-resource regions, and is becoming an increasingly urgent problem in low-resource regions, where incidence rates have been increasing by up to 5% per year [1]. Despite earlier radiological examination, complete surgical resection and aggressive chemotherapy, it is still a social dilemma. Research studies have shown relevance of neuroendocrine molecules in breast cancer development, such as substance P and its receptor, NK-1, which belongs to G protein coupled receptor [2,3]. Substance P is a member of neurokinin family. Pharmacological studies have confirmed NK-1 as the high affinity receptor of substance P. It is well known that substance P and NK-1 are widely expressed in neural and non-neural sources [4-11]. Moreover, substance P could mediate cell mitogenesis through NK-1 activation [7], and using specific NK-1 antagonists (such as CP-96345, C-99994) in breast cancer cell lines could blunt the autocrine and/or paracrine cell proliferation [2,3].

Two forms of NK-1 are reported in humans, full-length (NK1-FL) and truncated (NK1-Tr). The cytoplasmic end of NK1-Tr lacks 100 residues, a region that functions as the substrate for G protein-receptor kinase [12]. By in situ hybridization, the existence of NK-1 mRNA has been demonstrated in malignant breast tissue but not detected in benign tissue [2]. Western blots showed coexpression of NK1-Tr and NK1-FL in several different breast cancer cell lines, including T47D [3]. Moreover, Previous RT-PCR study showed T47D cells contain more abundant NK-1 and substance P than others [3]. Both NK1-Tr and NK1-FL can activate PKC through incorporating G proteins, which has been suggested as a potential cancer target [13,14]. Recently, the expression of NK-1 in human tumors has been investigated using immunohistochemistry [8]. In several cell types, tumor cells bear more NK-1 than normal cells. These findings suggest that NK-1 may serve as a specific factor involved in the development of breast cancer. However, it is unknown the exact cellular location of NK-1 in breast cancer cells. Although earlier in vitro studies have demonstrated that NK-1 antagonists could inhibit the growth of certain tumor cells in presence or absence of apoptosis [2,3,15-22], no study has been carried out on the antitumor action of specific NK-1 antagonist SR140333 in human breast cancer. Furthermore, it is also unclear whether the NK-1 specific agonist SMSP exerts proliferation promoting action or not in breast cancer cells.

Therefore, in this study, we first generated an immunohistochemical study to investigate the immunolocation of NK-1 on breast cancer tissues and T47D cell line. Then we examined the effect of SMSP and SR140333 on in vitro growth of human breast cancer cell line T47D and further detected whether the NK-1 receptor antagonist SR140333 produce apoptosis in this cell line. Our study may enable us to develop a potential therapeutic target for breast cancer therapy.

## Methods

### Reagents, antibodies, tumor tissues and cell line

Rabbit anti-NK1 (polyclonal antibody) was purchased from Millipore Corporation (USA). The anti-NK1 peptide was against the carboxyterminal tail of the NK-1 receptor, which corresponds to amino acids 393-407 of the NK1-FL receptor. Reagent A (Polymer enhancer), Reagent B (polymerized horseradish peroxidase-anti mouse/rabbit lgG), citrate buffer (pH = 6.0), normal non-immune goat serum (10%), and DAB were purchased from Maixin (Fuzhou, China). SMSP was obtained from Tocris (Avonmouth, UK). SR140333 was kindly provided by Sanofi-Aventis-Chilly-Mazarin. FBS, DMEM (high glucose), trypsin-EDTA (0.05% trypsin 0.53 mM EDTA) were purchased from Gibco (California, USA). MTT, DMSO and Hoechst33258 were purchased from Sigma (Saint Louis, USA). 25 cm^2 ^culture flakes, 96-well culture plates and 15 mL centrifuge tubes were purchased from Corning (New York, USA).

All breast tissues were obtained from Qingdao Municipal Hospital. The patients providing the tissues did not receive prior treatment with anticancer agents. The study was approved by the institutional review board of Qingdao University. The following tumors were investigated: infiltrating ductal carcinoma (n = 89), infiltrating lobular carcinoma (n = 14). The human breast cancer cell line T47D was purchased from Chinese Type Culture Collection (Shanghai, China). The T47D cells were seeded in 25 cm^2 ^culture flakes and maintained with DMEM supplemented with 10% FBS. The medium was renewed every two days and the cells were passaged by treatment with trypsin-EDTA on the six day after seeding. On the third day T47D cells entered exponential phase. Cells were incubated at 37°C in CO_2 _incubator (SHEL LAB, Oregon, USA) containing 5% CO_2_. All T47D cells were dissociated by treatment with trypsin-EDTA at 80-90% cell confluence and inoculated at a density of 10^5^cells/mL in 6-well plates which contained cover slips. The medium was renewed after two days and the cover slips were extracted on the fourth day, then the specimens were put into acetone (4°C) to fix for 15 minutes.

### Immunohistochemistry

All tissue specimens were fixed in formalin and embedded in paraffin. Seven-μm paraffin sections were cut and floated onto polylysine adhered slides. The sections were dewaxed in xylene and rinsed in alcohol and graded alcohol/water mixtures. The immunohistochemical staning was performed using Elivision™ plus two-step System. Briefly, all sections were incubated with 3% hydrogen peroxide for 15 minutes to block endogenous peroxidase activity at first. The sections were subsequently treated in a microwave oven twice for 6 minutes in citrate buffer at 600W to undergo antigen repairing. After blocking with goat serum for 30 minutes, rabbit anti-human NK-1 was applied on the sections at the dilution of 1: 700 for 90 minutes at room temperature. After rinsing, staining was performed with Reagent A and Reagent B subsequently. The color was developed by reacting with DAB. Sections were then counterstained with hematoxylin, dehydrated, cleared and coverslipped. The T47D cell specimens were also stained by the above procedures except antigen repairing. The specificity of immunolabelling was demonstrated by the absence of labelling for NK-1 receptors when the primary antibody was omitted. The benign breast tumors (fibroadenoma: n = 5 and adenosis: n = 6) are used for negative control. Pancreatic adenocarcinoma was used as positive control for the immunohistochemical study [23].

All specimens were observed by two investigators using an Olympus BX-51 microscope (Tokyo, Japan) Only the brown particles that were easily visible with a low power objective was categorized positive staining.

### Drug treatment

SMSP and SR140333 were dissolved in culture medium respectively to obtain experimental concentration. Different concentrations of SR140333 were evaluated in preliminary experiment to determine the 50% inhibition concentration (IC50) (unpublished data). In present study we performed various concentrations of SR140333 ranging from 10^-9^M to 10^-5^M to examine. In order to determine SMSP induced cell proliferation, different concentrations of SMSP (10^-10^M-10^-6^M) were evaluated. Furthermore, to learn whether SR140333 could counteract SMSP induced effect or not and at which concentration the counteract function would occur, we carried out competition experiments in which all T47D cells were treated using SMSP combined with various concentrations of SR140333. The most effective concentration of SMSP for this cell line was incubated 1 hour before the addition of SR140333.

### Proliferation assay

Cell proliferation was assessed using MTT assay. Cells were cultured in 96-well plates and the cell numbers were quantified using a coulter counter (Coulter Electronics, Inc., Hialeah, FL). Each well contained 2 × 10^4^cells in a total volume of 200 μL. The plate included blank wells (0 cells/mL), control wells (2 × 10^4^cells/0.2 Ml, untreated group), control wells with DMSO (no cells), control wells treated with SR140333 (10^-9^M-10^-5^M), control wells treated with SMSP (10^-10^M-10^-6^M) and control wells treated with SMSP (most effective concentration) combined with different concentrations of SR140333 (10^-9^M-10^-5^M). Drugs were added on day 3 (at exponential phase) and the assay was performed after 24 hours. For the proliferation assay, 20 μL MTT was added in each well. After 4 hour at 37°C supernatant was removed and 100 μL DMSO was added in each well. The optical density (OD) was detected in the microplate reader at 570 nm wavelength (Biotech Instruments, New York, USA). Each experimental condition (blank wells, control wells, and control wells treated with drugs) was assayed in duplicate and each study was repeated on at least three separate occasions. Representative data from each experiment are shown in this article.

### Growth study

T47D cells (2 × 10^5^cells/mL) were grown in 24-well tissue culture plates and each well containing 500 μL DMEM with 10% FBS. After 24 hours, medium was removed and fresh medium containing SMSP (the most effective concentration) or SR140333 (10^-5^M) was added at the same time (day 1). Control cells received only DMEM contained 10% FBS. On the subsequent five days, total cell counts were performed by a Coulter counter. Cell numbers determined by a Coulter counter were similar (less than 5% difference) to viable cell numbers determined by a dye (trypan blue) exclusion method using a hemocytometer.

### Hoechest33258 staining

In order to determine whether apoptosis is induced by the specific NK-1 antagonist SR140333, Hoechst33258 staining was performed. T47D cells were cultured in a 6-well plate using the cover slip culture method. On the third day SR140333 (10^-5^M) was added and 24 hours later all the cover slips were taken out. Control cells were treated only with culture medium. The cell samples were washed twice with PBS and fixed by incubation with glacial acetic acid/methanol mixture (glacial acetic acid: methanol = 1:3) for 30 minutes. Following washing in PBS, cells were incubated in 1 μg/mL Hoechst33258 solution for 10 minutes in the dark at 37°C. The cells were analyzed by a fluorescence microscope (Olympus BX-51, Tokyo, Japan).

### Statistical analysis

Statistical analysis was performed with SPSS 10.0 statistical software for Microsoft Windows. Values of proliferation assay and growth study were expressed as means ± SD. Data from the proliferation assay were analyzed using one-way ANOVA. The homogeneity of the variance was tested using the Levene test. If the variances were homogeneous, Fischer's least significant difference procedure for multiple comparisons with Bonferroni adjustment and Dunnett t tests were used. For data sets with non-homogeneous variances, the ANOVA test with T3 Dunnett post hoc analysis was applied. Data from growth study were analyzed using Dunnett t tests. We only considered the variances among different treating factors at the same day. The criterion for significance was p < 0.05 for all comparisons.

## Results

### Expression of NK-1 in breast cancer tissues and T47D cells

**Figure 1:**
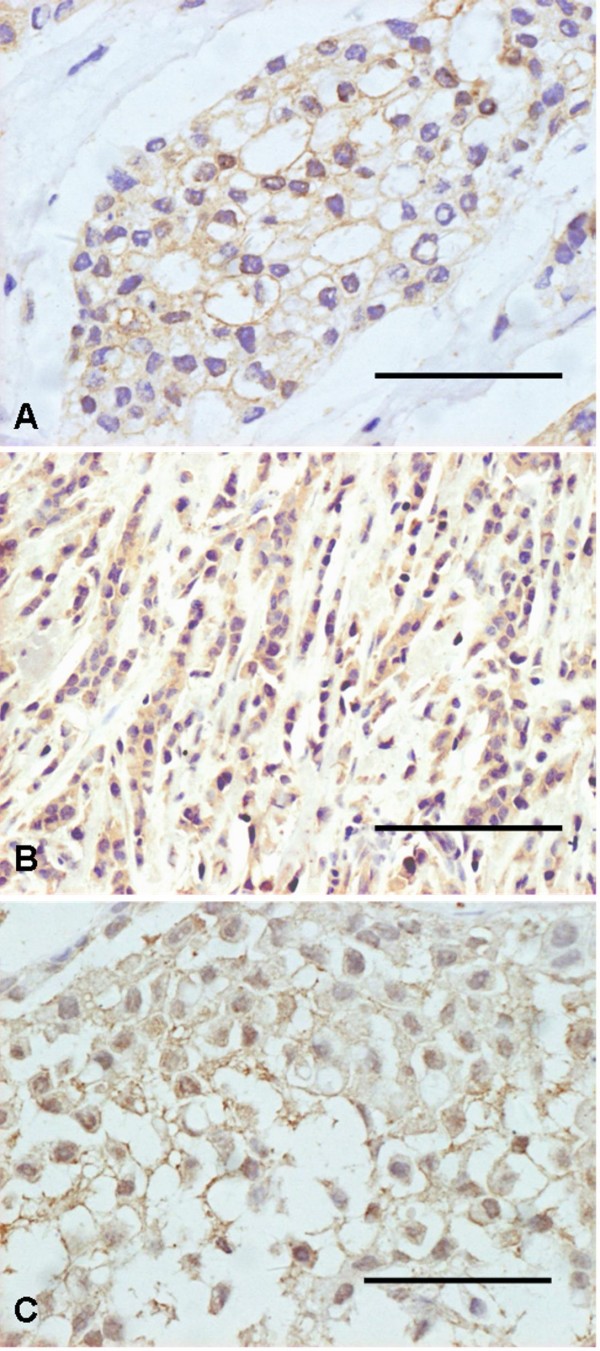
**Expression of NK-1 in Breast cancer and T47D cells**. A, Positive NK-1 receptor staining was present on nearly all tumor cells in infiltrating ductal cancer, and the plasma membranes were positively stained. B, Immunostaining of NK-1 receptor could also be observed in cytoplasma in infiltrating lobular cancer cells. C, The immunolabelling of NK-1 was located in membrane. Scale bars: A, C = 50 μm, B = 100 μm.

Prominent NK-1 immunostaining was detected in most malignant breast cancer tissues (infiltrating ductal carcinoma was 78/89 and infiltrating lobular carcinoma was 12/14, respectively) and T47D cells. The positively stained cells were widely distributed, and NK-1 receptors were present on nearly all breast cancer cells. The brown particles were frequently observed in plasma membrane and/or cytoplasma (Figure 1). The benign tumor tissues bear negative expression of NK-1.

### Effects of SMSP and SR140333 on the proliferation of T47D cells

**Figure 2:**
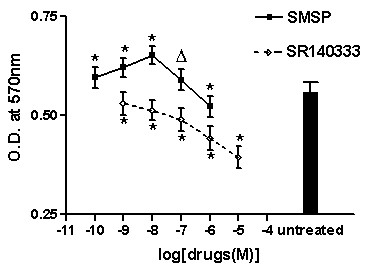
**Effect of different concentrations of [Sar9, Met(O2)11] substance P (SMSP) and SR140333 on proliferation of T47D cell line**. *p < 0.01; Δp < 0.05. Vertical bars indicate SD.

On the third day T47D cells entered exponential phase and different concentrations of SMSP was added. The results of MTT assay revealed that SMSP showed stimulatory effect at concentrations from 10^-10^M to 10^-7^M. Furthermore, at 10^-8^M SMSP exhibited the most effective stimulation manner. Instead, 10^-6^M of SMSP showed inhibitory effect as compared to the untreated group (Figure 2).

Proliferation inhibition of T47D cells by SR140333 was detected after the addition of increasing concentrations of the specific NK-1 antagonist. SR140333 showed the inhibitory effect in a dose dependent fashion at concentrations ranged from 10^-8^M to 10^-5^M, but 10^-9^M of SR140333 did not inhibit cell proliferation as compared to the untreated group (Figure 2).

**Figure 3:**
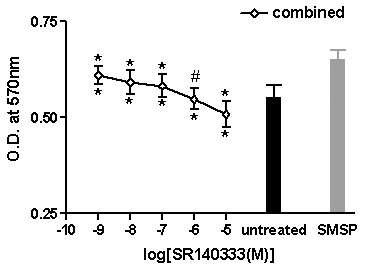
**Effect of SMSP (10^-8^M) combined with different concentrations of SR140333 (10^-9^M-10^-5^M) on proliferation of T47D cell line**. The asterisk below the bars indicates p value vs. SMSP group whereas that over the bars represents p value vs. untreated group. *p < 0.01; #no significance. Vertical bars indicate SD.

As 10^-8^M of SMSP exhibited the most effective stimulation manner, we took 10^-8^M as the most effective concentration to investigate. As compared with controls with SMSP alone, all cells showed proliferation inhibitory effect after administration of SMSP combined with various concentrations of SR140333. SR140333 inhibited the stimulatory effect of SMSP in a dose-dependent fashion. As compared with the untreated group, at 10^-6^M and 10^-5^M SR140333 could totally block 10^-8^M of SMSP induced stimulatory effect, and 10^-5^M of SR140333 showed inhibitory effect in the presence of 10^-8^M of SMSP. However, low concentrations of SR140333 (10^-9^M, 10^-8^M, and 10^-7^M) combined with 10^-8^M of SMSP still showed stimulatory effect. These results suggest SR140333 counteract SMSP induced proliferation in a dose dependent manner. Furthermore, SR140333 could block even reverse SMSP induced cell proliferation (Figure 3).

**Figure 4:**
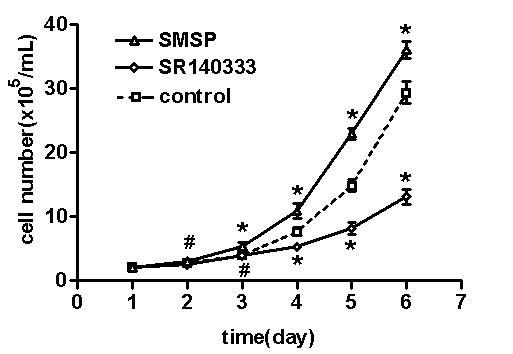
**Growth curve for T47D cell line in the presence of SMSP (10^-8^M) and SR140333 (10^-5^M) alone (evaluation by cell counting method)**. Both reagents were added respectively when the populations adhere to the flask. At different times, T47D cells were detached and then counted using a coulter counter. The results are shown as mean ± SD of four different experiments. Data of each day was analyzed by one-way ANOVA with Dunnett t test. *p < 0.01 vs. control; #no significance vs. control. Vertical bars indicate SD.

Compared with untreated group (control), cells treated with SMSP showed growth stimulatory effect from the third day while SR140333 showed growth inhibitory effect from the fourth day. In the successive five days after the administration of SR140333, growth rates of T47D cells were not reduced to zero, though (Figure 4).

### SR140333 induces apoptosis of T47D cells

**Figure 5:**
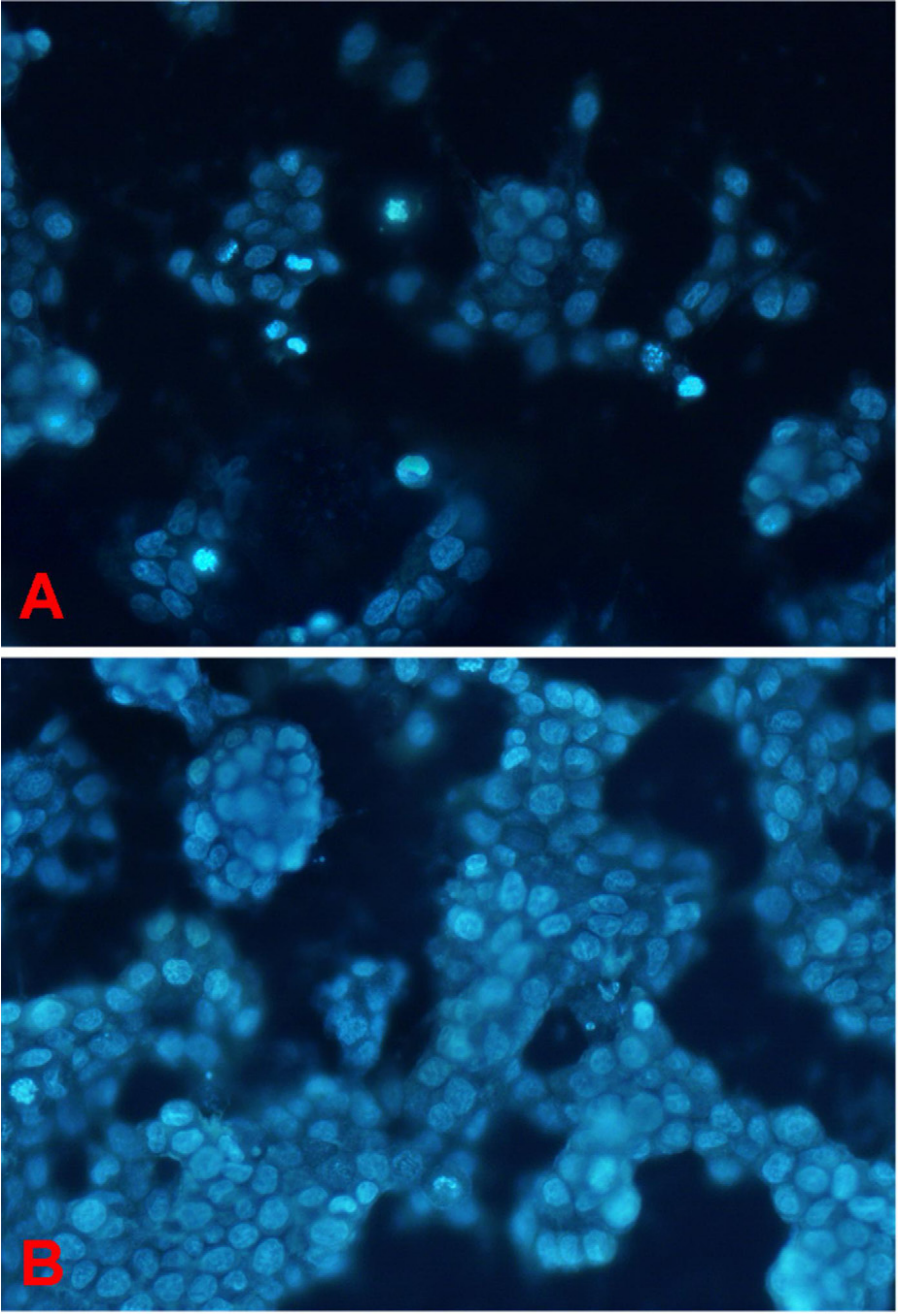
**Hoechst33258 fluorescent staining after SR140333 treatment (A, SR140333 treated cells; B, control)**. T47D cells were cultured in DMEM contained 10%FBS and SR140333 was added at logarithmic growth phase (on day 3, at about 30% cell confluences). We carried out Hoechst33258 staining on specimens obtained by the cover slip culture method. After treated with SR140333 for 24 h, T47D cells showed slower proliferation profile and visible apoptosis was detected by Hoechst33258.

Hoechst33258 staining revealed the presence of a great number of apoptotic cells after administration of SR140333 (Figure 5). Typical morphological change of apoptotic cells was easily observed, which showed characteristic of chromatin condensation and nuclear fragmentation. In fact, we observed a 25.58 ± 3.86 (SD) % of apoptotic cells after administration of SR140333 while only 7.85 ± 1.53 (SD) % in the untreated cells (p < 0.01).

## Discussion

Our present study has clearly demonstrated expression of NK-1 in breast cancer tissues and T47D cell line using immunohistochemical study. This result is in agreement with the previous study which demonstrated that NK-1 is increased in breast biopsies by in situ hybridization [2]. Moreover, previous study has shown that malignant breast tissues bear over-expression of substance P [2], indicating involvement of neuroendocrine mechanism in breast cancer development. NK-1 receptors in tumor cells increase the amount of mitotic signals for the tumor cell, counteracting the different apoptotic and/or pro-senescent pathways activated in the neoplastic cell population [24]. In breast cancers, increasing substance P could enhance the message transmitting through increasing NK-1; this may accelerate the proliferation process.

The increasing number of NK-1 in T47D cells leads us to investigate the role of NK-1 in tumor cell proliferation and growth. Therefore, we performed an in vitro study in which NK-1 receptors were activated or blocked by specific agonist SMSP or specific antagonist SR140333. The data of this study have shown, for the first time, that SMSP could stimulate the proliferation of breast cancer cell line T47D while SR140333 showed growth inhibitory effect. Further study by MTT assay has shown that SR140333 counteracted SMSP induced proliferation of T47D cells in vitro. These results suggest that the downstream signal transduction following NK-1 activation is significant for breast cancer development. It is known that substance P stimulates mitogenesis by activating NK-1 receptors in several neoplastic cell types [25,18,4-11]. Since we merely exerted SMSP not exogenous substance P in this study, the exact effect of substance P on breast cancer cell line is still unclear. As endogenous substance P exhibits high affinity to NK-1 in vivo [10,11], the present study suggests that NK-1 plays a central role in substance P related cell proliferation in T47D cells. Thus, we conclude that blocking NK-1 by SR140333 also could inhibit substance P induced cell proliferation in T47D cells. An observation cannot be explained was 10^-6^M of SMSP showed inhibitory effect, and the detail needs to be further studied.

The other finding in this study was the presence of visible apoptosis after administration of SR140333. This is consistent with earlier studies, in which the use of certain NK-1 antagonists inhibited the growth of other human breast cancer cell lines such as MDA-MB-231 and MDA-MB-468 [26,27]. It was speculated that this finding was induced by a signal transduction pathway for apoptosis [7,20,28,29]. In addition, the blockade of NK-1 could inhibit both DNA synthesis and cell proliferation by the mitogen-activated protein kinase (MAPK) pathway [25]. However, in the presence of CP-96345 or C-99994, which belongs to NK-1 antagonist, no apoptotic cells but only inhibitory effect was observed in human breast cancer cell line T47D [2,3]. The authors think the reason is that the cell cycle remained in the G_2 _phase [2]. Probably this different power action could be related with the different affinity for the NK-1 and with the expression of the amount of NK-1 receptors in the different tumor cells [30]. Moreover, previous studies have demonstrated that in the great majority of malignant tumors, NK-1 receptors were found on intra- and peritumoral blood vessels [6,23]. This finding indicated that NK-1 may serve as a preferred target for cancer therapy, which could mediate vasodilatation and mitogenesis. In fact, our unpublished immunohistochemical study has demonstrated the expression of NK-1 on both intratumoral and peritumoral blood vessels. Therefore, targeting NK-1 using SR140333 could decrease both nutrition supply and signal transduction.

It is well known that cell growth is regulated by various growth factors through their specific receptor linked various signal-transduction pathways [31]. A peptide growth factor may act through different receptors coupled to different post-receptor signal-transduction pathways [32] or the same receptor for a given peptide growth factor may be coupled to different post-receptor signal-transduction pathways by crosstalk [33]. T47D cells contain estrogen receptors (ER), and the ER dimer binds either directly to DNA at an estrogen response element or tethers to other bound transcription factors, thereby altering the transcription of estrogen sensitive genes [34] Although most ER is in the nucleus, a population resides in the cytoplasm and/or membrane, available for cross talk with other cytoplasmic/membrane-associated signaling molecules, such as shc. Because ER itself has no kinase activity, phosphorylation must occur through another molecule that associates with ER or is activated by the receptor. The activation of NK-1 induces releasing of G-protein βγ subunits, and the latter recruit components of the ras-dependent cascade, such as shc, grb2, and src, leading to the activation of raf-1 and MAPK [35]. Shc binds to ER and src in T47D cells, leading to estradiol-induced activation of the MAPK pathway [36]. Therefore, the estradiol-induced nongenomic signaling pathway can also be activated by downstream of NK-1 pathway. As most ER is in nucleus, genomic signaling pathway is more important than nongenomic pathway. We speculate blockade of NK-1 only cut estradiol-mediated MAPK pathway. At present, it is still unclear whether SR140333 could counteract estradiol induced T47D's proliferation or not. The blockade of NK-1 by SR140333 could only break off one of many kinds of receptor related cell proliferation. Thus, only slower growth rate was observed and the growth rate was not reduced to zero (Figure 2) after administration of antagonist SR140333.

## Conclusions

We have demonstrated the presence of NK-1 in breast cancer using immunohistochemistry. We also demonstrated the stimulatory effect of SMSP and inhibitory effect of SR1403333 on human breast cell line T47D. As only T47D cell line was bring into the present study, the effect of SR140333 on other cell lines is still not clear. Our observations indicate NK-1 may serve as a novel marker and target of breast cancer to study in the future.

## Abbreviations

SMSP: [Sar9, Met(O2)11]substance P; NK-1: neurokinin-1; PKC: protein kinase C; DAB: diaminobenzidine; FBS: Fetal bovine sera; DMEM: Dulbecco's Modified Eagle's Medium; MTT: 3-(4,5-Dimethylthiazol-2-yl)-2,5-diphenyltetrazolium; DMSO: dimethylsulfoxide; ER: estrogen receptor.

## Competing interests

The authors declare that they have no competing interests.

## Authors' contributions

WQH carried out the cell culture, drug treatment, MTT assay, and drafted the manuscript. JGW carried out the growth study and Hoechst 33258 staining and statistical analysis. LC carried out the immunohistochemical study. HJW collected tumor tissues. HC conceived of the study, and participated in its design and coordination and helped to draft the manuscript. All authors read and approved the final manuscript.

## References

[B1] International Agency for Research on Cancer World Cancer Report 2008 2008 Lyon

[B2] Singh D Joshi DD Hameed M Qian J Gascón P Maloof PB Mosenthal A Rameshwar P Increased expression of preprotachykinin-I and neurokinin receptors in human breast cancer cells: implications for bone marrow metastasis Proc Natl Acad Sci USA 2000 97 388 393 10.1073/pnas.97.1.388 10618428 PMC26673

[B3] Patel HJ Ramkissoon SH Patel PS Rameshwar P Transformation of breast cells by truncated neurokinin-1 receptor is secondary to activation by preprotachykinin-A peptides Proc Natl Acad Sci USA 2005 102 17436 17441 10.1073/pnas.0506351102 16291810 PMC1297665

[B4] Grotzer MA Janss AJ Fung KM Sutton LN Zhao H Trojanowski JQ Rorke LB Phillips PC Abundance of apoptotic neoplastic cells in diagnostic biopsy samples is not a prognostic factor in childhood primitive neuroectodermal tumors of the central nervous system J Pediatr Hematol Oncol 2001 23 25 29 10.1097/00043426-200101000-00007 11196266

[B5] Heppeler A Froidevaux S Eberle AN Maecke HR Receptor targeting for tumor localisation and therapy with radiopeptides Curr Med Chem 2000 7 971 994 10911025 10.2174/0929867003374516

[B6] Hennig IM Laissue JA Horisberger U Reubi JC Substance-P receptors in human primary neoplasms: tumoral and vascular localization Int J Cancer 1995 61 786 792 10.1002/ijc.2910610608 7790112

[B7] Esteban F Muñoz M González-Moles MA Rosso M A role for substance P in cancer promotion and progression: a mechanism to counteract intracellular death signals following oncogene activation or DNA damage Cancer Metastasis Rev 2006 25 137 145 10.1007/s10555-006-8161-9 16680578

[B8] Schulz S Stumm R Rocken C Mawrin C Schulz S Immunolocalization of full-length NK1 tachykinin receptors in human tumors J Histochem Cytochem 2006 54 1015 1020 10.1369/jhc.6A6966.2006 16651388

[B9] Pinto FM Almeida TA Hernandez M Devillier P Advenier C Candenas ML mRNA expression of tachykinins and tachykinin receptors in different human tissues Eur J Pharmacol 2004 494 233 239 10.1016/j.ejphar.2004.05.016 15212980

[B10] Beaujouan JC Torrens Y Saffroy M Kemel ML Glowinski J A 25 year adventure in the field of tachykinins Peptides 2004 25 339 357 10.1016/j.peptides.2004.02.011 15134859

[B11] Pennefather JN Lecci A Candenas ML Patak E Pinto FM Maggi CA Tachykinins and tachykinin receptors: a growing family Life Sci 2004 74 1445 1463 10.1016/j.lfs.2003.09.039 14729395

[B12] Fong TM Anderson SA Yu H Huang RR Strader CD Differential activation of intracellular effector by two isoforms of human neurokinin-1 receptor Mol Pharmacol 1992 41 24 30 1310144

[B13] Alblas J van Etten I Moolenaar WH Truncated, desensitization-defective neurokinin receptors mediate sustained MAP kinase activation, cell growth and transformation by a Ras-independent mechanism EMBO J 1996 15 3351 3360 8670836 PMC451898

[B14] Mackay HJ Twelves CJ Protein kinase C: a target for anticancer drugs? Endocr Relat Cancer 2003 10 389 396 10.1677/erc.0.0100389 14503915

[B15] Rosso M Robles-Frías MJ Coveñas R Salinas-Martín MV Muñoz M The NK-1 receptor is expressed in human primary gastric and colon adenocarcinomas and is involved in the antitumor action of L-733,060 and the mitogenic action of substance P on human gastrointestinal cancer cell lines Tumour Biol 2008 29 245 254 10.1159/000152942 18781096

[B16] Palma C Nardelli F Manzini S Correlation between binding characteristics and functional antagonism in human glioma cells by tachykinin NK1 receptor antagonists Eur J Pharmacol 1999 374 435 443 10.1016/S0014-2999(99)00334-9 10422788

[B17] Palma C Bigioni M Irrissuto C Nardelli F Maggi CA Manzini S Anti-tumour activity of tachykinin NK1 receptor antagonists on human glioma U373 MG xenograft Br J Cancer 2000 82 480 487 10.1054/bjoc.1999.0946 10646908 PMC2363296

[B18] Muñoz M Pérez A Rosso M Zamarriego C Rosso R Antitumoral action of the neurokinin-1 receptor antagonist L-733 060 on human melanoma cell lines Melanoma Res 2004 14 183 188 10.1097/01.cmr.0000129376.22141.a3 15179186

[B19] Muñoz M Rosso M Pérez A Coveñas R Rosso R Zamarriego C Piruat JI The NK1 receptor is involved in the antitumoural action of L-733,060 and in the mitogenic action of substance P on neuroblastoma and glioma cell lines Neuropeptides 2005 39 427 432 10.1016/j.npep.2005.03.004 15939468

[B20] Muñoz M Rosso M Coveñas R Montero I González-Moles MA Robles MJ Neurokinin-1 receptors located in human retinoblastoma cell lines: antitumor action of its antagonist, L-732,138 Invest Ophthalmol Vis Sci 2007 48 2775 2781 10.1167/iovs.05-1591 17525212

[B21] Muñoz M Rosso M Aguilar FJ González-Moles MA Redondo M Esteban F NK-1 receptor antagonists induce apoptosis and counteract substance P-related mitogenesis in human laryngeal cancer cell line HEp-2 Invest New Drugs 2008 26 111 118 10.1007/s10637-007-9087-y 17906845

[B22] González Moles MA Esteban F Ruiz-Avila I Gil Montoya JA Brener S Bascones-Martínez A Muñoz M A role for the substance P/NK-1 receptor complex in cell proliferation and apoptosis in oral lichen planus Oral Dis 2009 15 162 169 10.1111/j.1601-0825.2008.01504.x 19036058

[B23] Friess H Zhu Z Liard V Shi X Shrikhande SV Wang L Lieb K Korc M Palma C Zimmermann A Reubi JC Büchler MW Neurokinin-1 receptor expression and its potential effects on tumor growth in human pancreatic cancer Lab Invest 2003 83 731 742 12746482 10.1097/01.lab.0000067499.57309.f6

[B24] Payan DG Brewster DR Missirian-Bastian A Goetzl EJ Substance P recognition by a subset of human T lymphocytes J Clin Invest 1984 74 1532 1539 10.1172/JCI111567 6207205 PMC425324

[B25] Luo W Sharif TR Sharif M Substance P-induced mitogenesis in human astrocytoma cells correlates with activation of the mitogenactivated protein kinase signaling pathway Cancer Res 1996 56 4983 4991 8895754

[B26] Irrissuto C Maggi CA Goso C Role of NK-1 and NK-2 tachykinin receptor antagonism on the growth of human breast carcinoma cell line MDA-MB-231 Anticancer Drugs 2005 16 1083 1089 10.1097/00001813-200511000-00007 16222150

[B27] Lang K Drell TL Lindecke A Niggemann B Kaltschmidt C Zaenker KS Entschladen F Induction of a metastatogenic tumor cell type by neurotransmitters and its pharmacological inhibition by established drugs Int J Cancer 2004 112 231 238 10.1002/ijc.20410 15352035

[B28] Muñoz M Rosso M Coveñas R The NK-1 receptor is involved in the antitumoural action of L-733,060 and in the mitogenic action of substance P on human pancreatic cancer cell lines Lett Drug Des Discov 2006 3 323 329 10.2174/157018006777574168

[B29] Muñoz M Rosso M Coveñas R Fernandes JA NK-1 receptor antagonists as new anti-tumoural agents: action on human neuroblastoma cell lines Focus on neuroblastoma research 2007 New York: Nova Science 31 56

[B30] Muñoz M Rosso M Soult JA Coveñas R Yang AV Antitumoural action of neurokinin-1 receptor antagonists on human brain cancer cell lines Brain cancer: therapy and surgical intervention 2006 New York: Nova Science 45 75

[B31] Rozengurt E Neuropeptides as cellular growth factors: role of multiple signalling pathways Eur J Clin Invest 1991 21 123 134 10.1111/j.1365-2362.1991.tb01801.x 1647950

[B32] Ishizuka J Beauchamp RD Townsend CM Jr Greeley GH Jr Thompson JC Receptor-mediated autocrine growth-stimulatory effect of 5-hydroxytryptamine on cultured human pancreatic carcinoid cells J Cell Physiol 1992 150 1 7 10.1002/jcp.1041500102 1309821

[B33] Millar JBA Rozengurt E Bombesin enhancement of cAMP accumulation in Swiss 3T3 cells: evidence of a dual mechanism of action J Cell Physiol 1988 137 214 222 10.1002/jcp.1041370203 2848040

[B34] Carroll JS Brown M Estrogen receptor target gene: an evolving concept Mol Endocrinol 2006 20 1707 1714 10.1210/me.2005-0334 16396959

[B35] van Biesen T Hawes BE Raymond JR Luttrell LM Koch WJ Lefkowitz RJ G(o)-protein alpha-subunits activate mitogenactivated protein kinase via a novel protein kinase C-dependent mechanism J Biol Chem 1996 271 1266 1269 10.1074/jbc.271.3.1266 8576109

[B36] Zhang Z Kumar R Santen RJ Song RX The role of adapter protein Shc in estrogen non-genomic action Steroids 2004 69 523 529 10.1016/j.steroids.2004.05.012 15288764

